# Pharmacotherapy for obesity: are we ready to select, tailor and combine pharmacotherapy to achieve more ambitious goals?

**DOI:** 10.3389/fendo.2025.1569468

**Published:** 2025-06-18

**Authors:** Nele Steenackers, Julia Toumassian, Ellen Deleus, Ann Mertens, Matthias Lannoo, Sofia Pazmino, Amar Daniël Emanuel van Laar, Bart Van der Schueren, Roman Vangoitsenhoven

**Affiliations:** ^1^ Clinical and Experimental Endocrinology, Department of Chronic Diseases and Metabolism, KU Leuven, Leuven, Belgium; ^2^ Department of Nutrition and Movement Sciences, Research Institute of Nutrition and Translational Research in Metabolism (NUTRIM), Maastricht University, Maastricht, Netherlands; ^3^ Department of Abdominal Surgery, University Hospitals, Leuven, Belgium; ^4^ Department of Endocrinology, University Hospitals Leuven, Leuven, Belgium

**Keywords:** obesity, obesity pharmacotherapy, precision medicine, personalized treatment, combination therapy, weight management, weight loss

## Abstract

Recent advancements in obesity pharmacotherapy have seen the approval of novel agents, like glucagon-like peptide-1 receptor agonist and dual agonists, offering unprecedented efficacy for obesity management. However, treatment outcomes remain highly variable, necessitating a more personalized approach to pharmacotherapy tailored to individual profiles. This review evaluates the current landscape of obesity pharmacotherapy, while exploring factors influencing variability in treatment response including early response predictors, genetic markers, and physiological traits. Additionally, the potential of combining treatment modalities and some emerging drugs are highlighted. Finally, a stepwise algorithm is proposed for personalized obesity treatment, integrating comorbidities, phenotypes, and responses to medication, paving the way for more effective and efficient obesity management.

## Introduction

Obesity is a chronic disease with significant public health implications (1, 2). Characterized by an excessive accumulation of fat that impairs health, obesity increases the risk of developing serious comorbidities, such as cardiovascular disease, type 2 diabetes, musculoskeletal disorders, certain cancers, and psychological disorders (3, 4). The physical health consequences together with the stigma surrounding obesity negatively impacts the quality of life and reduces life expectancy (5). Economically, obesity poses an important financial burden, accounting for approximately 7% of the European healthcare budgets, and an estimated € 70 billion annually in the European Union, including healthcare costs and lost productivity (6). Furthermore, the effects of obesity surpass individual health and societal costs by influencing future generations through potential epigenetic changes (7).

Despite the increasing prevalence and predictions suggesting that a quarter of the world’s population will suffer from obesity by 2035 (8), a detailed understanding of the disease’s pathophysiology and management remains limited (1, 9). Although once thought, the origin of obesity is much more complex than simply an accumulation of excess calories and sedentary behavior (1, 9). A dynamic interplay of environmental, genetic, psychological, and physiological factors can lead to a central dysregulation of energy balance, causing a tendency to promote weight gain and impeding weight loss (1, 9). This complexity underscores the need for a paradigm shift from solely focusing on weight loss to managing a healthy energy homeostasis.

Addressing obesity is challenging but not insurmountable as several effective treatments are available. Lifestyle modification, including medical nutrition therapy and increased physical activity, is the key pillar in the treatment of obesity (10, 11). While these approaches might achieve the desired weight loss of at least 5%, sustaining the loss can be challenging (12). Metabolic/bariatric surgery has for a long time been the only weight loss intervention with long-term weight loss (on average 30%) and associated health benefits (13). However, the widespread application is affected by limited accessibility, resource requirements, potential complications, and a patients resistance to undergo a surgical procedure (14, 15). With lessons learned from bariatric surgery, a great deal of research has been devoted towards the development of pharmacotherapy as a non-invasive, safe and effective treatment alternative for obesity treatment (16). After decades of weight loss medication with poor safety profiles, such as with sibutramine or rimonabant, recent progress in pharmacotherapy has led to the approval of a new generation of anti-obesity medications, starting with liraglutide (once-daily) followed by semaglutide (once-weekly), by the Food and Drug Administration (FDA) and the European Medicines Agency (EMA) (**Table 1**) (17). These therapies, recommended for individuals with a body mass index (BMI) ≥ 30 kg/m² or for individuals with a BMI ≥ 27 kg/m^2^ and obesity-related comorbidities such as hypertension, type 2 diabetes and sleep apnea, mark the beginning of a new era in obesity treatment (11). Medications like semaglutide have achieved double digit weight loss percentages (>10%), previously seen only with surgical interventions. However, large interindividual variability in weight loss outcomes highlights the complexity of obesity and its treatment (18). This heterogeneity underscores the need for a more personalized approach to pharmacotherapy, as “one-size-fits-all” strategies are unlikely to succeed. Currently, there are no guidelines to assist clinicians in selecting pharmacotherapy tailored to a patient’s clinical profile. This review provides an overview of available pharmacotherapies, identifies knowledge gaps in treatment guidelines, and propose strategies for personalized patient selection, sequence and combination approaches to improve treatment outcomes.

**Table 1:** Currently approved and available weight loss drugs.

Name	Mechanism of action	Dose	FDA/EMA approved	% weight loss from baseline	Administration	Common Adverse Effects	Ref.
Orlistat	Lipase inhibitor	120 mg 3x daily	FDA 1999, EMA 1998	-10.2% (at 52 weeks)	PO	Steatorrhea, flatulence, fecal urgency	(22, 28, 36)
Phentermine/ Topiramate	GABA agonist/Carbonic anhydrase inhibitor	15/92 mg once-daily	FDA 2012	-9.3% (at 108 weeks)	PO	Paresthesia, suicidal ideation, sleep disorders, increased heart rate	(37)
Naltrexone/ Bupropion	Opioid antagonist/catecholamine reuptake inhibitor	16 mg/180 mg 2x daily	FDA 2014, EMA 2015	-6.1% (at 56 weeks)	PO	Nausea, seizures, hypertension, glaucoma, xerostomia, depression	(29, 35)
Liraglutide	GLP-1 receptor agonist	3 mg once-daily	FDA 2014, EMA 2015	−8% (at 52 weeks)	SC	Nausea, vomiting	(21)
Semaglutide	GLP-1 receptor agonist	2.4 mg once-weekly	FDA 2021, EMA 2022	-14.9% (at 68 weeks)	SC	Nausea, vomiting	(20, 38)
Tirzepatide	Dual GLP-1 and GIP receptor agonist	15 mg once-weekly	FDA 2023, EMA 2023	-20.9% (at 72 weeks)	SC	Nausea, diarrhea, constipation	(23, 39)

EMA, European Medicines Agency; FDA, Food and Drug Administration (United States); GIP, Glucose-dependent insulinotropic polypeptide; GLP-1, Glucagon-like peptide-1; PO, Oral administration; SC, Subcutaneous injection.

## Search strategy and selection criteria

References for this review were identified through searches of PubMed for articles published from January, 1947, to May 2025. A combination of search terms was applied pertinent to the topic of obesity pharmacotherapy including “obesity pharmacotherapy”, “GLP-1 receptor agonist”, “semaglutide”, “tirzepatide”, “liraglutide”, “naltrexone/bupropion”, “phentermine/topiramate”, “orlistat”, “retatrutide”, “survodutide”, “weight loss medications”, “Body Mass Index”, “BMI”, “weight management”, “weight loss” and “obesity”. Articles were screened based on their relevance for the current review. Reference list cited in those articles were reviewed to identify if some studies have not been captured. Only articles published in English were included.

## Currently approved pharmacotherapy

**Figure 1:**
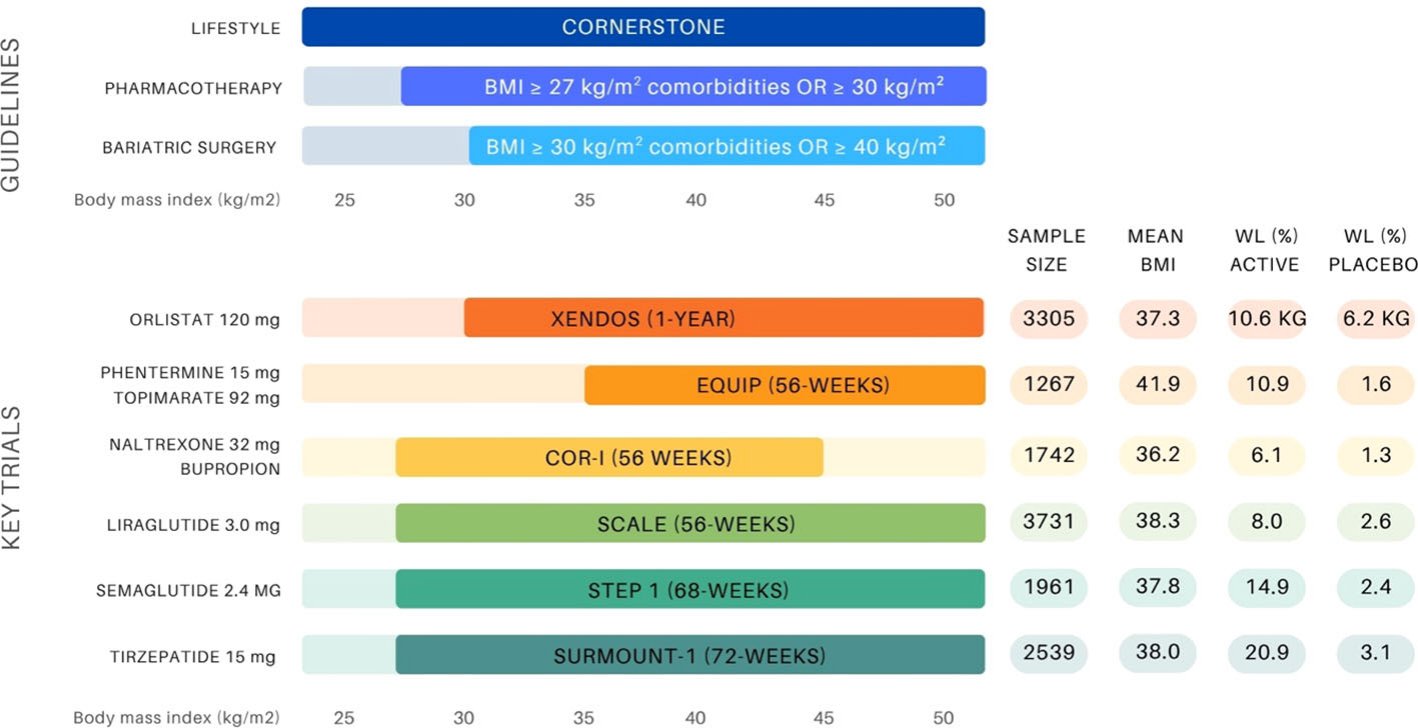
Overview of BMI-based treatment recommendations for obesity management and landmark anti-obesity drug trials (10, 11, 14, 19–21, 23, 25, 34, 35). The upper panel presents guideline thresholds for lifestyle intervention, pharmacotherapy (BMI ≥27 kg/m² with comorbidities or ≥30 kg/m²), and bariatric surgery (BMI ≥35–40 kg/m² depending on comorbidity status). The lower panel displays key clinical trials supporting the approval of six anti-obesity agents, indicating sample size, mean baseline BMI (entire group or active group), and weight loss outcomes (%WL unless otherwise stated) in both the active and placebo arms.

Body mass index (BMI) thresholds for obesity interventions vary depending on the treatment modality, as outlined in **Figure 1**. Pharmacotherapy is indicated for patients with a BMI ≥30 kg/m² or ≥27 kg/m² with comorbidities. Currently, the FDA has approved six medications, and the EMA has approved five medications that are available for chronic weight management (**Table 1**). The approvals are based on robust clinical trial data demonstrating their efficacy and safety with indications typically targeting a specific BMI category (10, 11, 19–27).

Orlistat, the earliest approved anti-obesity medication, remains available (Brand name ‘Xenical’ produced by Roche; Brand name ‘Alli’ produced by GlaxoSmithkline). It acts as a gastric and pancreatic lipase inhibitor, reducing the absorption of dietary fat by preventing the lipase-catalyzed breakdown in the gastrointestinal tract, leading to weight loss (22, 28). While it has been widely used, the gastrointestinal side effects including steatorrhea and malabsorption often lead to treatment discontinuation due to their (socially) intolerable nature (28). The next approved drugs include the centrally acting agents, namely naltrexone extended release (ER)/bupropion ER (Brand name ‘Contrave’ produced by Orexigen Therapeutics Inc, brand name ‘Mysimba’ in Europe) and phentermine/topiramate ER (Brand name ‘Qsymia’ produced by Vivus, Inc). The first combination includes naltrexone, an opioid receptor antagonist, traditionally used to treat alcohol/opioid dependence and bupropion, a norepinephrine and dopamine reuptake inhibitor, indicated as antidepressant/smoking cessation aid (29). The second combination includes phentermine, a sympathomimetic amine, suppresses appetite that is potentiated by topiramate, which modulates satiety through GABA receptor activation (30). Both therapies achieve similar weight loss results as orlistat, but these combinations might induce other side effects like nausea, irritability, depression, suicidal ideation and cardiovascular complications (i.e. phentermine), limiting their suitability for certain patient populations (29, 30). Initially developed for type 2 diabetes management, two incretin analogues have been approved that were previously indicated for diabetes management, namely liraglutide (Brand name ‘Saxenda’ produced by Novo Nordisk) and semaglutide (Brand name ‘Wegovy’ produced by Novo Nordisk). By stimulating the glucagon-like-peptide-1 (GLP-1) receptor, they stimulate insulin secretion, suppress appetite, increase satiety and slow gastric emptying, thereby reducing calorie intake and potentially changing food preferences (31). Although both drugs operate through the same mechanism, the more recently approved semaglutide has a much larger weight loss effect (20).

Currently, naltrexone/bupropion and phentermine/topiramate are the only approved combination therapies, designed to synergistically target two distinct pathways to induce weight loss (26). However, dual GLP1 and glucose-dependent insulinotropic peptide (GIP) receptor agonists are refining the pharmacological landscape. These novel pharmacological strategies, such as tirzepatide, were initially developed to address glycemic management, but significantly impact weight, metabolic status and cardiovascular outcomes. By activating two complementary pathways, tirzepatide offers greater efficacy than GLP-1 receptor agonists alone (23). Being FDA-approved for diabetes management first in 2022 (Brand name ‘Mounjaro’ produced by Eli Lilly), tirzepatide received subsequent FDA approval under a Fast Track designation in 2023 (Brand name ‘Zepbound’ produced by Eli Lily) (32). Clinical trials have demonstrated weight reductions averaging between 15-20% depending on dosage, outcomes comparable to the outcomes historically only achieved with bariatric surgery (16). While these medications demonstrate significant efficacy, long-term data on cardiovascular outcomes and mortality remains limited for most (33). Although GLP-1 receptor agonists have shown favorable cardiovascular profiles with reductions in major adverse cardiovascular events, robust evidence linking these therapies to reduced all-cause mortality is still under investigation​. Future research must focus on addressing these gaps to fully establish the role of pharmacotherapy in improving long-term complications and survival rates.

## (Early) responders and non-responders for weight loss

**Figure 2:**
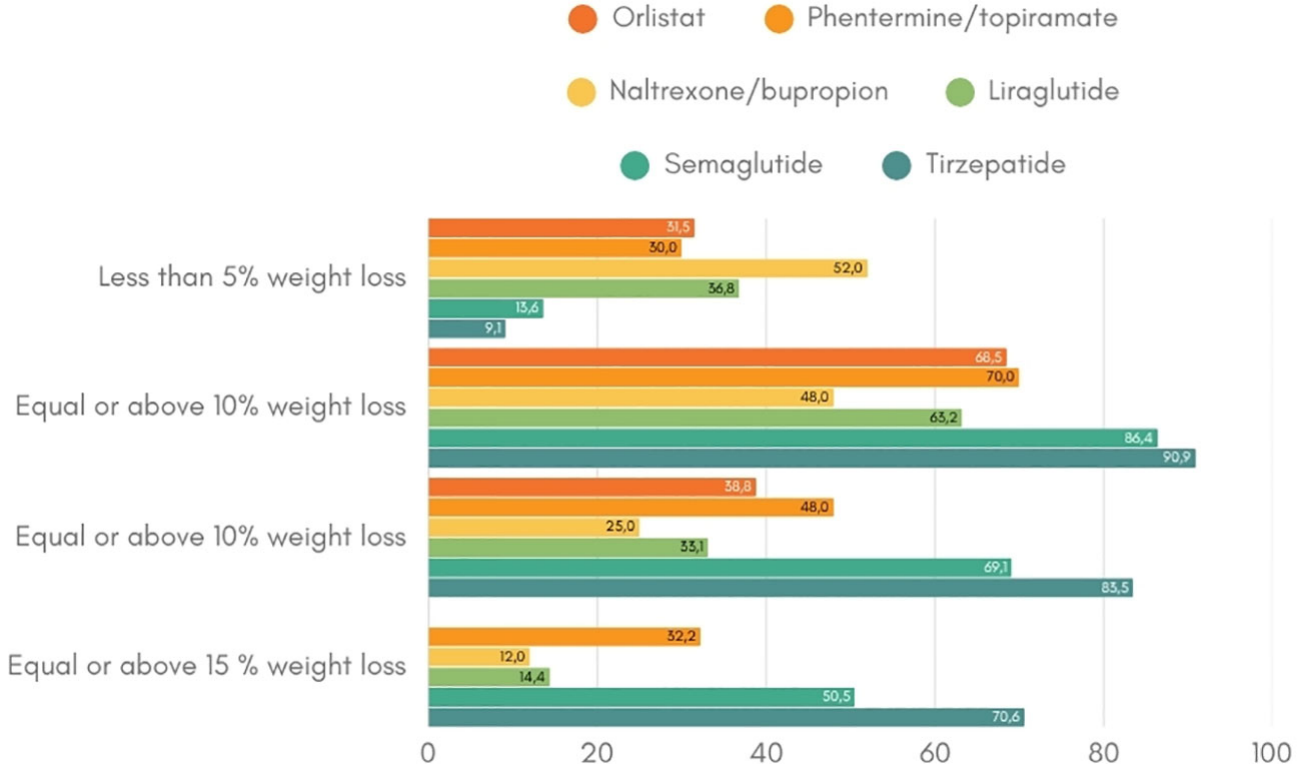
Weight loss response per anti-obesity drug (19–21, 23, 25, 34, 35). This figure indicates the percentage of patients that achieve the weight loss targets of at least 5%, 10% and 15% per anti-obesity medication.

The variability in response to anti-obesity interventions remains a significant and poorly understood aspect of weight management (40). Across all obesity treatment modalities – lifestyle interventions, pharmacotherapy, and bariatric surgery – patients can be classified into either ‘responders’ or ‘non-responders’ based on their weight loss outcomes following treatment. The definition of a ‘responder’ varies slightly between clinical studies but generally refers to a weight loss of 3-5% within the first three to six months of treatment (34, 41–44). This heterogeneity in treatment responses suggest the existence of underlying determinants – both biological and environmental – that influence efficacy among patients (**Figure 2**). Notably, non-response rates vary across pharmacotherapy ranging from 9 to 52%, with naltrexone/bupropion having the highest proportion of non-responders. In contrast, the newer incretin-based therapies, such as GLP-1 receptor agonist and dual GLP-1/GIP receptor agonists, show lower non-response rates. For instance, preliminary findings from the phase 2 trial of retatrutide, a triple hormone receptor agonist, reported a 100% response rate of more than 5% weight loss for the dosages of 8 or 12 mg weekly over 48 weeks (45). This reflects the improved efficacy and response rates associated with these newer drug classes. Real-world data will have to proof the durability in treatment response and persistence in drug adherence.

To date, little is known about specific predictive factors for treatment response, especially for pharmacotherapy (40). However, one well-established predictor of long-term weight loss is an early response to treatment (34, 41, 42). Substantial weight loss during the initial phase of treatment correlates with greater weight loss after 12 months, while minimal early weight loss signals a poor likelihood of response over time (34, 41, 42). This principle is integrated in the European guidelines for obesity treatment, which recommend discontinuing anti-obesity medication if patients lose less than 5% of their weight (or less than 3% in patients with diabetes) within the first three months of treatment (11). Patients who meet or exceed the threshold are considered ‘early responders’, while those who do not are considered ‘non-responders’, warranting a change in therapeutic approach. While early weight loss is a valuable predictor, it does not fully explain the factors determining individual response to treatment. Continued research is needed to improve response prediction and optimize personalized pharmacotherapy.

Research has indicated that the presence of type 2 diabetes significantly impacts weight loss response (46). Over the past three decades, while glycemic control in diabetes has steadily improved, the average BMI of patients with diabetes has also increased (47). Research using older generations of anti-obesity medication indicated that individuals with both obesity and diabetes faced greater challenges in achieving weight loss (46). A *post-hoc* analysis of pooled data from the SCALE Obesity and Prediabetes and SCALE Diabetes trial demonstrated notable differences in the proportion of ‘early responders’ and ‘early non-responders’ to liraglutide. Over one-third of patients with type 2 diabetes were classified as non-responders compared to less than a quarter of patients without diabetes (without type 2 diabetes: 77.3% responders and 22.7% non-responders; with type 2 diabetes: 62.7% responders and 37.3% non-responders) (42). Furthermore, among responders, the weight reduction was generally lower for patients with type 2 diabetes compared to those without diabetes (-8.5% versus -10.8 after 56 weeks). Although the full mechanism underlying this difference remains unclear, several contributing factors have been proposed. Both insulin-resistance, which hampers fat oxidation, and the use of certain antidiabetic medications like insulin and sulfonylurea promote weight gain and might counteract the effects of anti-obesity medication (48–50). Moreover, intensive glycemic control itself has been linked with weight gain (51).

However, the development of newer drugs like tirzepatide offer hope for improved outcomes (52, 53). The SURMOUNT-2 trial, which investigated tirzepatide for obesity treatment in people with type 2 diabetes, reported a mean body weight reduction of 12.8% and 14.7% for weekly doses of 10 and 15 mg, respectively, after 72 weeks (53). This represents the highest weight reduction achieved in a phase 3 trial for this population to date (53, 54). These results suggest that dual agonists are promising obesity treatment options for managing obesity in patients with diabetes. In addition, the current EASD guidelines include bariatric surgery as a treatment option, and weight management is increasingly recognized as a key factor in the choice of antidiabetic medication within the choice of treatment (55, 56).

To explore the role of genetic predictors, recent research pooled data from seven major lifestyle intervention trials (NUGENOB, DIOGenes, Look AHEAD, Diabetes Prevention Program, Diabetes Prevention Study, DIETFITS, PREDIMED-PLUS). By doing so, a polygenic score comprising 59 single nucleotide polymorphisms was identified to partially explain variability in weight loss among white participants, expressed through a composite measure of waist circumference, waist-to-hip ratio and BMI. However, this was not observed in African American participants. Although the effect was small and clinically significant, the findings highlight the potential role of genetic variants associated with central adiposity in obesity management. Further investigation is required to determine whether this polygenic score influence pharmacotherapy outcomes (57).

Regarding currently available anti-obesity medication (**Table 1**), more research on genetic influences on treatment response is emerging. For instance, a Taq1A polymorphism related to striatal dopamine D2 receptor density has been shown to affect the weight loss response to naltrexone/bupropion (43). This pilot study found that patients carrying the A1+ allele were more likely to respond and achieve higher weight loss (A1+ genotype: 5.9 ± 3.2%; A1-genotype: 4.2 ± 4.2%; P=0.03) (43). Although the sample size was rather small, these findings present an interesting perspective for advancing personalized medicine. Similarly, variability in weight loss responses to GLP-1 receptor agonists has raised interest in pharmacogenomic studies in people with type 2 diabetes. A genome-wide analysis revealed that variation in HbA1C reduction with GLP-1 receptor agonist treatment was associated with a common genetic variation in GLP1R (rs6923761G→A (Gly168Ser)) and a rare variant in *ARRB1* (rs140226575G→A (Thr370Met)) (58). Moreover, patients with type 2 diabetes carrying the variant allele (A) of the rs6923761 GLP-1 R polymorphism demonstrated greater reductions in BMI, weight, and fat mass with liraglutide treatment (59). While these findings were obtained using type 2 diabetes treatment, it raises the possibility that variants in genes encoding GLP-1 receptor might affect weight loss response in people with obesity alone.

A pilot study investigating GLP-1 receptor genes in patients with obesity has provided further insight (60). This study investigated two polymorphisms – rs6923761 (p.Gly168Ser) and rs10305420 (p.Pro7Leu) – in 57 women with obesity and polycystic ovarian syndrome (PCOS). Significant distinctions were found between responders and non (or poor) responders with responders achieving greater weight loss of (7.38 ± 1.74 kg vs 2.11 ± 2.17kg, respectively). Furthermore, individuals carrying at least one rs10305420 allele experienced significantly less weight loss compared to those with wild-type alleles (60). While those with at least one rs6923761 allele had superior outcomes, consistent with the findings from type 2 diabetes studies (58, 59). Despite these promising associations, the clinical application of pharmacogenomics in obesity pharmacotherapy remains premature. Most findings to date stem from pilot studies with limited replication across independent cohorts. For now, insufficient data limits the current clinical utility.

Beyond type 2 diabetes and genetic variants, some physiological markers have been identified as predictors of weight loss response to anti-obesity medication. For example, gastric emptying half-time has been shown to correlate with weight loss outcomes. Delayed gastric emptying at five weeks is related with greater weight loss at 16 weeks of liraglutide treatment (61). Additionally, a proof-of-concept randomized controlled study found that baseline food intake was associated with the weight loss response at two weeks of treatment with central acting phentermine/topiramate treatment (62). Overall, weight loss response appears to be a multifactorial phenomenon influences by biological, environmental and psychosocial components, many of which remain to be fully elucidated.

## Combining lines of treatment

To date, evidence supporting add-on therapy for obesity remains limited and rather speculative. A few trials explored the additional effect of adding pharmacotherapy with other weight loss management strategies, such as lifestyle interventions and metabolic surgery. However, most studies have focused on specific patient subgroups, limiting the generalizability of the findings to the broader population of individuals with obesity. In the GRAVITAS randomized controlled trial, liraglutide has been studied as an add-on therapy for patients with persistent type 2 diabetes following bariatric surgery (63). While the primary objective was the change in HbA1c levels, secondary endpoints included weight loss. The study demonstrated significant additional weight loss of -4.23 kg compared to the control group after 26 weeks, irrespective of the type of bariatric surgery. However, the trial only included patients with both type 2 diabetes and obesity, leaving questions about its applicability to the general population with obesity. In 2019, Wharton et al. conducted an observational study to investigate add-on liraglutide 3.0 mg therapy for insufficient weight loss or weight regain after bariatric surgery, irrespective of diabetes presence (64). The results showed average weight loss of -5.5% ± 6.2% with liraglutide independent of the type of surgery performed. However, the lack of control group limits the strength of these findings to some extent. A prospective, randomized controlled trial is needed to confirm liraglutide’s efficacy as adjunct therapy after bariatric surgery.

Liraglutide has also been studied in combination with a very low calory diet in the SCALE maintenance study (65). In this randomized controlled trial, patients who already lost equal or more than 5% of their initial weight during a low-calorie diet run-in were randomly assigned to liraglutide or placebo. The liraglutide group not only maintained their weight loss better (81.4% versus 48.9%) but also achieved additional weight loss compared to the control group, with 50.5% versus 21.8% reaching at least 5% weight loss, respectively. Furthermore, improvements in certain cardiovascular risk factors were observed (65). However, since the trial only included a subgroup of patients who responded well to an initial low-calorie diet, it remains unclear whether liraglutide with diet would benefit patients who do not respond to dietary interventions alone. Additionally, the study by Lundgren et al. examined the effect of liraglutide with exercise on weight loss (66). The findings revealed that combining pharmacotherapy with exercise resulted in superior weight loss compared to either of the interventions alone. Moreover, post-treatment analysis demonstrated improved weight loss maintenance with combination therapy after treatment termination (67). Significantly more patients who underwent both supervised exercise and pharmacotherapy maintained at least 10% weight loss one year after treatment cessation (45%), compared to those who received only exercise (29%), only liraglutide (16%) or placebo (10%) (67).

Together, these studies suggest that a combinative approach to obesity management may improve outcomes both in terms of the magnitude of weight loss and its long-term maintenance. However, the current body of evidence remains insufficient to draw definitive conclusions. Larger, well-controlled add-on trials are necessary to substantiate the benefits of combining pharmacotherapy with other treatment modalities. In addition to combining pharmacotherapy with lifestyle or surgical interventions, there is potential benefit of combining different pharmacotherapies to target multiple pathways or organs. Existing combination therapies, such as phentermine/topiramate and naltrexone/bupropion, as well as the dual agonist tirzepatide, represent steps in this direction. However, there is a lack of evidence for using multiple pharmacotherapies simultaneously, as studies investigating this approach are currently unavailable.

## Tailor treatment to each patient

Obesity is a heterogenous disease with multifactorial origins, a diverse patient population, and significant variability in treatment response. Despite this complexity, current treatments predominantly focus on BMI thresholds with little consideration for individual phenotypes or response variability (10, 11). However, BMI alone is insufficient to describe or predict health status or mortality risk in individuals (68). The Edmonton Obesity Staging System, which accounts for comorbidities and functional status, offers better mortality predictions without relying on BMI or adiposity (68). This underscores the need for stratified treatment strategies tailored to patient profiles. Given its epidemiology, its diverse presentations, and suboptimal treatment outcomes for some patients, it is becoming evident that a ‘one-size-fits-all’ approach is inadequate when discussing obesity treatment (69, 70). Precision medicine, which incorporates genetics, environment, metabolites and other individual factors, holds promise for optimizing treatment outcomes (71, 72). A clustering approach – using comorbidities, or phenotypes, – offers a pragmatic first step towards tailoring treatment (73, 74). By aligning choices of anti-obesity drug with their specific mechanism of action towards patient specific factors, better treatment responses can be achieved (75).

### Comorbidities

BMI is currently the primary criterion for prescribing weight loss medications with thresholds of ≥30 kg/m² for general obesity and ≥27 kg/m² for those with comorbidities. However, tailoring therapy based on the specific comorbidities can maximize therapeutic benefits rather than addressing each condition separately (10, 11). Evidence suggest that certain anti-obesity medications not only target the excess weight but also address underlying comorbidities (74).

Type 2 diabetes, a common obesity-related comorbidity, significantly improves with weight loss of at least 15% (76). GLP-1 receptor agonists - liraglutide, semaglutide and tirzepatide - were initially developed as antidiabetic agents due to their ability to stimulate insulin secretion in a glucose-dependent manner (77–79). The LEAD trial (liraglutide) and the SUSTAIN trial (semaglutide) demonstrated that both drugs effectively reduce body weight, glycemia and HbA1c in patients with type 2 diabetes (77, 78). Tirzepatide has shown superior glycemic control, reducing fasting glucose by 15 to 20 mg/dL and HbA1C by 1% more than semaglutide and insulin Glargine (80–82). In the SURMOUNT-1 trial, tirzepatide not only produced substantial and sustained weight loss over 176 weeks in individuals with obesity and prediabetes, but also significantly delayed the onset of type 2 diabetes compared to placebo. The incidence of type 2 diabetes was markedly lower in the tirzepatide groups versus placebo at week 176 (1.3% vs. 13.3%; HR: 0.07 (95% CI: 0.0 to 0.1), P<0.001), and this benefit persisted after a 17-week off-treatment period (HR: 0.12 (95% CI: 0.1 to 0.2), P<0.001) (83). These findings support tirzepatide’s potential to serve not only as a weight loss agent, but also as a preventive strategy for type 2 diabetes in high-risk populations. Beyond these, naltrexone/bupropion significantly lowered HbA1c levels in patients with obesity and type 2 diabetes in the COR-DM trial, although no significant changes were observed in fasting glucose or insulin (84).

Cardiovascular disease is another major obesity-related comorbidity. Evidence regarding naltrexone/bupropion’s cardiovascular effects remain inconclusive, following publication of the results for the prematurely terminated LIGHT outcomes study. The trial was halted after the original commercial sponsor inappropriately released preliminary findings of a confidential early analysis. While the 25% interim analysis suggested a potential reduction in MACE (HR: 0.59; 95% CI: 0.39-0.90), subsequent data did not confirm this benefit. The 50% analysis showed a neutral effect (HR: 0.88; 99.7% CI: 0.57–1.34) (85). In contrast, GLP-1 agonists demonstrated protective cardiovascular effects (86–89). The SELECT trial, published in 2023, was the first to demonstrate that semaglutide 2.4 mg reduces the risk of major cardiovascular events (MACE) by 20% in patients with obesity without diabetes (86). This establishes semaglutide as the first weight-loss medication to show cardiovascular benefit independent of glycemic effects (86). In contrast, earlier trials like the LEADER (liraglutide) and SUSTAIN-6 (semaglutide 1.0 mg) trial enrolled people with type 2 diabetes and high cardiovascular risk. Both trials showed significant reductions in the incidence of cardiovascular death, nonfatal myocardial infarction, and nonfatal stroke (87, 90). PIONEER-6, evaluating oral semaglutide, confirmed non-inferiority for cardiovascular outcomes in patients with type 2 diabetes (88). In patients with obesity and heart failure with a preserved ejection fraction included in the STEP-HFpEF trial, semaglutide significantly reduced symptoms, improved physical limitations, and enhanced exercise functions compared to placebo (89). The cardioprotective effect of GLP-1 agonists are likely mediated by reductions in visceral adipose tissue (88). Tirzepatide has shown remarkable promise in improving cardiovascular outcomes in patients with obesity and heart failure with preserved ejection fraction (HFpEF). In the SUMMIT trial (91), Tirzepatide significantly reduced the risk of a composite endpoint of cardiovascular death or worsening heart failure events compared to placebo. Death from cardiovascular causes or a worsening heart-failure event occurred in 9.9% of the tirzepatide group versus 15.3% of the placebo group. Beyond reducing adverse cardiovascular outcomes, tirzepatide led to substantial improvements in health status, as reflected by a 6.9-point greater increase in the Kansas City Cardiomyopathy Questionnaire clinical summary score over 52 weeks. Patients also experienced significant weight loss (−13.9% vs. −2.2%) and a marked reduction in systemic inflammation, as indicated by decreased high-sensitivity C-reactive protein levels. These findings highlight tirzepatide’s multifaceted cardiometabolic benefits, suggesting that it may be a transformative therapy for managing both obesity and HFpEF by targeting excess adiposity, systemic inflammation, and cardiovascular risk (91). Despite these promising results, a limitation in the field remains the lack of long-term cardiovascular outcomes data for newer agents. To address this, the SURMOUNT-MMO trial is currently underway, investigating tirzepatide’s effects on cardiovascular morbidity and mortality in adults with obesity, irrespective of diabetes status (92). Similarly, SYNCHRONIZE-CVOT (survodutide), a glucagon/GLP-1 receptor co-agonist, is being evaluated in a large-scale randomized controlled trial to assess its cardiovascular safety and efficacy in people with obesity (93). These trials will be pivotal in establishing the long-term benefits and safety of next-generation anti-obesity medications beyond weight loss alone.

In addition to being a serious comorbidity, obstructive sleep apnea syndrome (OSAS) exerts a negative influence on weight loss capacity by impairing muscle energy metabolism, reducing exercise capacity, and altering ghrelin levels that regulate hunger (94). The SCALE sleep study demonstrated that liraglutide significantly improves OSAS, by reducing the apnea-hypopnea index by 12.2 apnea events per hour compared to 6.1 events per hour in the control group (95). This effect may be linked to a reduced GLP1 receptor response observed in individuals with OSAS. In the SURMOUNT-OSA phase 3 trials, tirzepatide markedly reduced the apnea–hypopnea index by up to 29.3 events per hour over 52 weeks compared to minimal reductions with placebo in people with obesity treated with positive airway pressure (estimated treatment difference: 23.8 events per hour (95% CI: −29.6 to −17.9), P<0.001). This improvement was accompanied by substantial weight loss, alongside significant decreases in hypoxic burden, high-sensitivity C-reactive protein levels, and systolic blood pressure. Additionally, patients reported better sleep quality and reduced sleep-related impairment, which in turn can impact weight gain (96). These findings highlight dual benefit in effectively managing obesity-driven sleep apnea, offering a promising pharmacological alternative to traditional mechanical therapies such as positive airway pressure.

Obesity is a significant contributor to reduced fertility and infertility in women (97–100). Women with obesity typically experience poorer reproductive outcomes including longer time to conception, and an increased risk of miscarriage (97–101). One of the leading causes of infertility in women of reproductive age is polycystic ovary syndrome (PCOS), a condition often exacerbated by obesity (97). Weight reduction plays a crucial role in managing infertility and other PCOS-related symptoms, such as hyperlipidemia, hyperandrogenism, decreased insulin sensitivity, and hypertension (102–104). Evidence suggest that PCOS pathogenesis may be linked to alterations in GLP-1 receptors, supporting the use of GLP-1 receptor agonists as a potential treatment in these cases (105, 106). A randomized controlled trial involving women with PCOS and obesity demonstrated that treatment with liraglutide significantly improved reproductive markers including increased sex hormone-binding globulin, decreased free testosterone and ovarian volume, and improved bleeding ratio (107). Although evidence on pregnancy outcomes with GLP-1 receptor agonists is still emerging, two studies offer promising insights. In one trial, women randomized to receive either Exenatide or Metformin, followed by Metformin alone, achieved a natural pregnancy rate over twice as high in the exenatide group compared to the metformin group (43.60% vs 18.70%) (108). Similarly, a small pilot study showed that combining liraglutide with Metformin before conception significantly increased *in vitro* fertilization pregnancy rates compared to Metformin alone (109).

Metabolic dysfunction-associated steatotic liver disease (MASLD) is a prevalent metabolic disorder that significantly increases the risk of developing cardiovascular disease, type 2 diabetes, chronic kidney disease, liver cirrhosis, and both intra and extrahepatic cancers (110). Addressing MASLD is critical in managing obesity-related complications due to its strong association with metabolic dysfunction. Evidence from a pooled *post hoc* analysis suggests that naltrexone/bupropion may positively influence liver health by improving the liver fibrosis index and reducing alanine aminotransferase levels (111). However, further research is needed to confirm these findings. GLP-1 receptor agonists might be effective in both reducing cardiovascular disease, and hepatic health (112). Specifically, all three approved GLP-1 agonists - liraglutide, semaglutide, and Exenatide – have shown significant reductions in liver fat content (113–115). The LEAN study highlighted liraglutide’s impact on liver fibrosis progression, where 9% of patients treated with liraglutide experienced fibrosis progression compared to 36% in the placebo group (113). A detailed overview of pharmacological strategies targeting MASLD in the context of obesity management is provided elsewhere (116).

In addition, GLP-1 receptor agonists are effective in managing reduced satiety a common issue in patients with obesity (61). These medications prolong satiety by acting on the central nervous system, and delaying gastric emptying, a physiological response of which the magnitude may serve as predictive marker for future weight loss (61, 117).

Depression and obesity are highly intertwined with each condition increasing the risk of developing the other (118). For patients with coexisting depression and obesity, naltrexone/bupropion can be considered first-line pharmacotherapy due to the antidepressant properties of bupropion, which functions as a noradrenalin and dopamine reuptake inhibitor (119). Supporting this approach, a *post hoc* analysis of clinical trials indicated that patients with obesity treated with naltrexone/bupropion exhibited lower rates of depression (119). Moreover, some antidepressants and antipsychotic medications are associated with weight gain, potentially worsening obesity. In such cases, transitioning to bupropion either alone or in combination with naltrexone could be a suitable option for managing both mood disorders and obesity, provided this approach is agreed upon with the treating psychiatrist or general practitioner.

Smoking addiction is another critical condition that exacerbates obesity-related health risks, significantly increasing the likelihood of developing or reinforcing cardiovascular disease, type 2 diabetes, and chronic obstructive pulmonary disease (120). Addressing smoking cessation is essential in comprehensive obesity management (120). Notably, bupropion was originally approved for smoking cessation, making naltrexone/bupropion a good choice for patients dealing with both obesity and nicotine dependence (121). In addition, naltrexone/bupropion effectively targets emotional eating due to its dual action on mood regulation, appetite control, and craving suppression (73, 122–125). This combination has shown to reduce the frequency and magnitude of food cravings by reducing the central food reward effect to food stimuli (26, 35). Moreover, a combination of liraglutide and intensive behavioral therapy significantly improved binge eating behaviors and eating disorder psychopathology at 24 weeks, but these benefits attenuated over time (126).

Beyond managing common comorbidities, there is emerging evidence that anti-obesity medications may also improve conditions less frequently associated with obesity such as neurodegenerative diseases and osteoarthritis. Among these, GLP-1 receptor agonists have gained particular attention for its potential neuroprotective effects that may offer therapeutic benefits in Parkinson’s disease (125). This is currently being investigated in a phase 2 clinical trial (127).

### Obesity phenotypes

Beyond BMI and comorbidities, a more nuanced classification of obesity can be achieved by considering a patient’s metabolic profile, behavioral traits, and physiological characteristics. Recognizing this complexity, Acosta et al. proposed a phenotypic classification system that stratifies patients into four different subtypes driven by specific pathophysiological and behavioral mechanisms, aiming to tailor therapy accordingly (73). In their study, patients were evaluated based on multiple parameters including body composition, resting energy expenditure, satiety, satiation, eating behavior, emotional affect, and physical activity. From this comprehensive assessment, four obesity phenotypes were identified including hungry brain characterized by abnormal satiation, emotional hunger characterized by hedonic eating, hungry gut characterized by abnormal satiety, and slow burn characterized by a decreased metabolic rate. Pharmacological treatments were selected per phenotype, targeting the main driving factor (phentermine/topiramate for hungry brain, naltrexone/bupropion for emotional hunger, liraglutide for hungry gut, and phentermine for slow burn) (73). This phenotype-driven strategy demonstrated promising results. Patients, who received treatment tailored to their phenotype, achieved significantly greater weight loss after 12 months compared to those receiving standard care (15.9% vs 9.0%, respectively) (73). The concept of this phenotype-driven strategy is rather new. Not all patients fit neatly into one phenotype, and additional undiscovered factors may further refine this model (128). Despite these limitations, phenotype-based treatment holds substantial potential for improving patient outcomes by increasing treatment efficacy, sustainability of weight loss, and optimized use of pharmacotherapy.

Beyond tailoring pharmacotherapy to comorbidities and phenotypes, clinicians must also account for body composition changes that extend beyond fat mass. While the efficacy of anti-obesity medications is relatively well documented, emerging evidence indicates potential adverse effects on muscle and bone mass. GLP-1 receptor agonists, in particular, may lead to disproportionate loss of lean mass, especially in older adults (129). This has implications for frailty, fracture risk, and long-term functional status. A secondary analysis of a randomized clinical trial further supports these concerns, showing that liraglutide treatment alone significantly reduced hip and lumbar spine bone mineral density (BMD) compared with exercise or placebo. However, the combination of liraglutide with moderate- to vigorous-intensity exercise preserved BMD at all clinically relevant sites (66). Until more data are available, clinicians are advised to monitor muscle mass and functional performance especially in at-risk populations.

### Future prospects – dose titration and personalized nutrition

Emerging research highlights several promising avenues for enhancing the personalization and effectiveness of obesity pharmacotherapy. While the standard recommended dose of liraglutide is 3.0 mg daily, research suggests that maximum dosage is not always necessary to achieve the same amount of weight loss (130). A retrospective study evaluating real-world liraglutide use implemented weekly up-titration up to 3.0 mg/day (130). However, many patients were unable to tolerate the full dose due to side effects and maintained using lower dose of 1.2, 1.8 or 2.4 mg/day. Surprisingly, patients on these lower doses experienced weight loss comparable to those on the full dose of 3.0 mg, with average weight reductions of 7.4, 7.8, 9.0 and 8.0 kg for dosages of 1.2, 1.8, 2.4 and 3.0 mg, respectively (130). This observation implies that not all patients need to take on the highest dose to have the beneficial effects of liraglutide. Some individuals may have a heightened sensitivity to liraglutide’s effects, potentially due to genetic variations in the GLP-1 receptor or other unknown factors. These findings emphasize the need for individualized dose titration to identify the minimal effective dose per patient, maximizing therapeutic outcomes while minimizing side effects and reducing medication costs. Dose titration can be a new form of tailored treatment, serving as a valuable strategy in precision medicine. However, as this evidence is currently limited to liraglutide, further research is warranted to explore wither similar dose-response variability exists for other anti-obesity medications. Additionally, studies are needed to identify predictive factors that can guide clinicians in tailoring dosages to individual patient profiles.

Another promising option for individualizing obesity is the integration of personalized nutrition. A study by Zeevi et al. demonstrated that tailoring dietary recommendations to individual patients significantly improved postprandial glycemic responses in individuals with diabetes (131). This approach utilized multifaceted data, including blood biomarkers, CGM-derived features, gut microbiome composition, anthropometric measurements, food intake records, and lifestyle questionnaires to develop customized dietary plans (131). Due to the interrelatedness of obesity and type 2 diabetes, a similar strategy in obesity management could enhance the effectiveness of lifestyle interventions. While precision nutrition holds significant potential, its detailed exploration is beyond the scope of this review and has been reviewed elsewhere (132).

Expanding on earlier discussions, genetic profiling holds potential for advancing personalized obesity treatment. Variations in genes encoding drug targets, such as GLP-1 receptor polymorphisms, have been linked in clinical research to differential responses to medications like liraglutide and naltrexone/bupropion’s. Incorporating pharmacogenomic testing could enable clinicians to predict which patients will respond best to specific medications, allowing for more effective and individualized treatment strategies. For now, insufficient data limits its current clinical utility.

## Developing an algorithm for personalized obesity treatment

**Figure 3:**
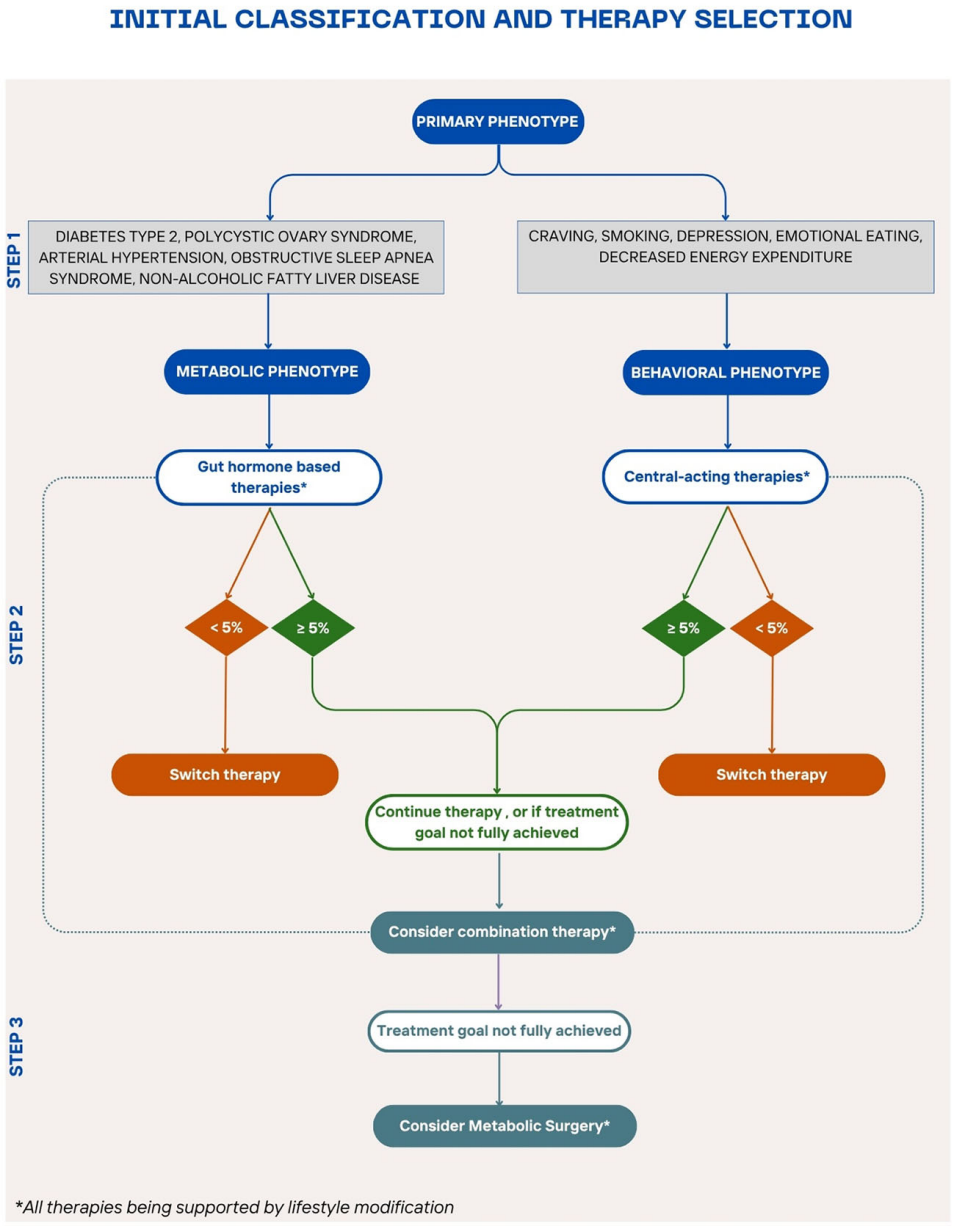
Stepwise algorithm for personalized obesity pharmacotherapy A proposed treatment algorithm integrating patient phenotypes, comorbidities, and treatment responses to guide personalized obesity management for patients who do not meet the criteria for metabolic surgery or have contraindications or not open to this option. The algorithm begins with lifestyle modification as the foundation, followed by phenotype-driven pharmacotherapy selection—gut hormone-based therapy for metabolic comorbidities and centrally acting therapy for behavioral/psychological factors. Treatment response is evaluated after 3 months to determine the need for therapy adjustment, combination treatment, or escalation to bariatric surgery for non-responders .

To achieve more precise and effective obesity management tailored to individual patient’s needs, the integration of various clinical and behavioral factors into a structured treatment pathway is essential. For this approach to be practical for clinicians, a streamlined, evidence-based guiding tool is required. A useful starting point is to draw parallels between obesity and other chronic, multifactorial diseases such as type 2 diabetes and arterial hypertension (133, 134). These conditions share complex etiologies and require long-term management strategies. In the management of type 2 diabetes, the American Diabetes Association and the European Association for the Study of Diabetes emphasize a holistic patient-centered approach that considers comorbidities like obesity, chronic kidney disease, hypertension and cardiovascular disease when selecting treatments (56). This paradigm could serve as a model for obesity care, moving towards a personalized, stepwise treatment plan similar to established hypertension guidelines, where first-line treatments are selected based on patient profiles and therapeutic needs (135). However, a major challenge in translating such frameworks to obesity lies in the lack of universally accepted, objective stratifiers limiting precise classification. Therefore, we emphasize a risk-factor-based escalation model, where comorbidities, treatment response, and functional limitations guide progression through treatment steps. Inspired by this approach and integrating factors previously discussed, we propose a preliminary algorithm for obesity management. Although this model does not fully encompass all aspects of precision medicine, it serves as a practical foundation for individualized treatment. Each transition in our proposed algorithm is grounded in trial evidence, guideline thresholds, and comparative safety/efficacy profiles. The first step involves stratifying patients based on their predominant obesity phenotype, while recognizing overlap and individual variability. These are partially informed by the subtypes identified by Acosta et al. and partially by the health of the patient (73) (**Figure 3**). This distinction classifies patients into two dominant categories: a metabolic phenotype, characterized by comorbidities such as type 2 diabetes, hypertension, obstructive sleep apnea, and polycystic ovary syndrome), and a central/behavioral phenotype, dominated by behavioral and psychological factors such as increased cravings, emotional eating, smoking addiction, and depression. Regardless of phenotype, lifestyle modification, including dietary changes and increased physical activity, remains the foundational treatment (10, 11). Based on their clinical profile, patients can receive gut hormone-based therapy (i.e. semaglutide, liraglutide, or tirzepatide) or central acting therapy (i.e. naltrexone/bupropion, or phentermine/topiramate) as a first-choice treatment. Patients with metabolic risk factors or established comorbidities benefit most from gut hormone-based therapies (62, 87, 88, 95, 97, 136). Although most evidence supports semaglutide and liraglutide, tirzepatide, as a dual GLP-1 and GIP receptor agonist, likely offers similar benefits, though further research is warranted (137). Patients struggling with obesity and concurrent smoking addiction, depression, or emotional eating, should begin treatment with centrally acting medication such as naltrexone/bupropion or phentermine/topiramate (119, 121).

When initiating pharmacotherapy, patients should be closely monitored to assess early response to pharmacotherapy. Dose titration is critical as some patients may be more sensitive to lower dosages and can achieve effective weight loss without reaching the maximum recommended dose (130). After three months, evaluate if the patient has achieved a weight loss of 5% or more in non-diabetic patients, or 3% or more in diabetic patients in line with the European guidelines for obesity management (11). Responders should continue their current therapy, while non-responders should transition to an alternative pharmacotherapy or initiate combination therapy. While combination pharmacotherapy is promising, there are currently no formal guidelines, and robust clinical trials are needed to validate this strategy. If weight loss goals remain unmet after trying alternative or combination pharmacotherapy, escalation to more invasive treatments such as metabolic bariatric surgery should be considered. Bariatric procedures have demonstrated superior long-term weight loss outcomes but are typically reserved for patients with severe obesity or those who do not achieve clinically relevant improvements with less invasive interventions. Overall, our algorithm is intended not as a rigid decision tree, but as a flexible, risk-stratified framework that supports clinical reasoning and individualized care. Undoubtedly, further clinical research is essential to refine this model, validate predictive markers of treatment response, and establish clear guidelines for combination therapies.

## Future treatments and remaining research gaps

Ongoing research in obesity pharmacotherapy continues to yield promising developments, with two treatments particularly relevant for their potential impact. Retatrutide, a triple agonist targeting the GLP-1, GIP, and glucagon receptors, represents a significant advancement beyond dual agonists like tirzepatide. In phase II trials, retatrutide demonstrated a mean weight loss of 24.2% at 48 weeks at the highest dosage (12 mg daily), compared to a 2.1% reduction in the placebo group, exceeding the impressive results achieved by tirzepatide (45, 138). Its safety profile thus far aligns with other approved incretin-based therapies, suggesting it could be a transformative option in the future for obesity management (136).

In addition to retatrutide, a growing pipeline of emerging compounds includes other multi-agonists such as Survodutide (GLP-1/glucagon co-agonist), and Cagrilintide (a long-acting amylin analogue), all currently being evaluated in advanced clinical trials (139). While each agent targets different hormonal pathways, these candidates reflect a broader strategy to enhance efficacy and address the limitations of existing therapies. Despite the efficacy of incretin-based therapies, their reliance on subcutaneous administration might limit convenience for some patients. The development of oral formulations addresses this challenge. An oral form of semaglutide, Rybelsus, is approved for diabetes but requires administration 30 minutes before meals for efficacy (140, 141). An oral GLP-1 receptor agonist, orfoglipron, has demonstrated promising results in phase II trials for obesity, with weight loss ranging from 9.4% to 14.7% over 36 weeks, comparable to other injectable GLP-1 analogues (142). While tirzepatide already exists as an incretin-based drug with superior weight loss outcomes, an oral formulation like orfoglipron could may increase treatment acceptability and adherence. A detailed overview of emerging pharmacotherapies is provided elsewhere (139).

Despite the substantial progress in obesity pharmacotherapy, several critical gaps persist. First, the long-term safety, and effects on mortality remain incompletely established for some agents. Second, post-marketing data reveal that real-world adherence and persistence are lower than in clinical trials, often due to gastrointestinal side effects, treatment fatigue, or cost-related barriers (143–145). Third, additional real-world effectiveness data are urgently needed to complement trial findings and assess outcomes in more diverse populations (146). In addition, real-world data on cost-effectiveness and optimal treatment duration are limited. For instance, one study reported that although tirzepatide would avert 45 609 obesity cases (95% uncertainty interval (UI): 45 092 - 46 126) per 100 000 individuals and semaglutide would avert 32 087 cases (95% UI: 31 292 - 32 882) per 100 000 individuals, their respective incremental cost-effectiveness ratios in the United States were $197 023 per QALY and $467 676 per QALY in the US. To reach the $100 000/QALY threshold, the current net prices would need to be reduced by 30.5% for tirzepatide and 81.9% for semaglutide. This raises questions regarding long-term economic sustainability of these therapies, if prices remain unaltered (147). Fourth, head-to-head comparative effectiveness studies remain scarce, which limits the available evidence to guide clinical decisions on optimal treatment sequencing, switching between therapies, and the potential benefits or risks of combination therap. Fifth, regulatory and reimbursement varies across countries and regions, affecting its widespread implementation (148). Finally, the integration of pharmacogenomics into treatment guidelines is not yet feasible due to insufficient clinical validation, although early data are promising. Addressing these unanswered questions should be a priority for future research.

## Conclusion

Obesity remains an increasing health problem worldwide. Its treatment goes on to be a complex issue, with a variety of factors influencing treatment outcomes. While metabolic bariatric surgery has for a long term been the only effective weight loss intervention for morbid obesity, newly developing pharmacotherapy are reaching comparable results, offering both an alternative and a complementary approach. Still, outcomes of treatment remain variable. While previous reviews have highlighted the promise of obesity pharmacotherapy, we outline different factors that influence patients’ response to treatment, explore the options of added benefits by using medication specifically selected and targeted to patients’ comorbidities and their personal phenotype. Furthermore, this review uniquely proposes a stepwise, phenotype-driven treatment algorithm and synthesizes evidence on dose titration, genetic markers of drug response, and combination strategies to advance individualized care. While more research is needed to strengthen this type of approach, it can be a step towards more effective and efficient obesity treatment.
