# Correction: Pharmacotherapy for obesity: are we ready to select, tailor and combine pharmacotherapy to achieve more ambitious goals?

**DOI:** 10.3389/fendo.2025.1656611

**Published:** 2025-10-02

**Authors:** Nele Steenackers, Julia Toumassian, Ellen Deleus, Ann Mertens, Matthias Lannoo, Sofia Pazmino, Amar Daniël Emanuel van Laar, Bart Van der Schueren, Roman Vangoitsenhoven

**Affiliations:** ^1^ Clinical and Experimental Endocrinology, Department of Chronic Diseases and Metabolism, KU Leuven, Leuven, Belgium; ^2^ Department of Nutrition and Movement Sciences, Research Institute of Nutrition and Translational Research in Metabolism (NUTRIM), Maastricht University, Maastricht, Netherlands; ^3^ Department of Abdominal Surgery, University Hospitals, Leuven, Belgium; ^4^ Department of Endocrinology, University Hospitals Leuven, Leuven, Belgium

**Keywords:** obesity, obesity pharmacotherapy, precision medicine, personalized treatment, combination therapy, weight management, weight loss

The figure captions were in the wrong order in the PDF version of this paper. Specifically, **Figure 1** was intended to be **Figure 3**. Its caption was at **Figure 2**. **Figure 2** was intended to be **Figure 1**. Its caption was at **Figure 3**. **Figure 3** was intended to be **Figure 2**. Its caption was at **Figure 1**. The corrected captions of **Figures 1**–**3** appear below.

**Figure 1:**
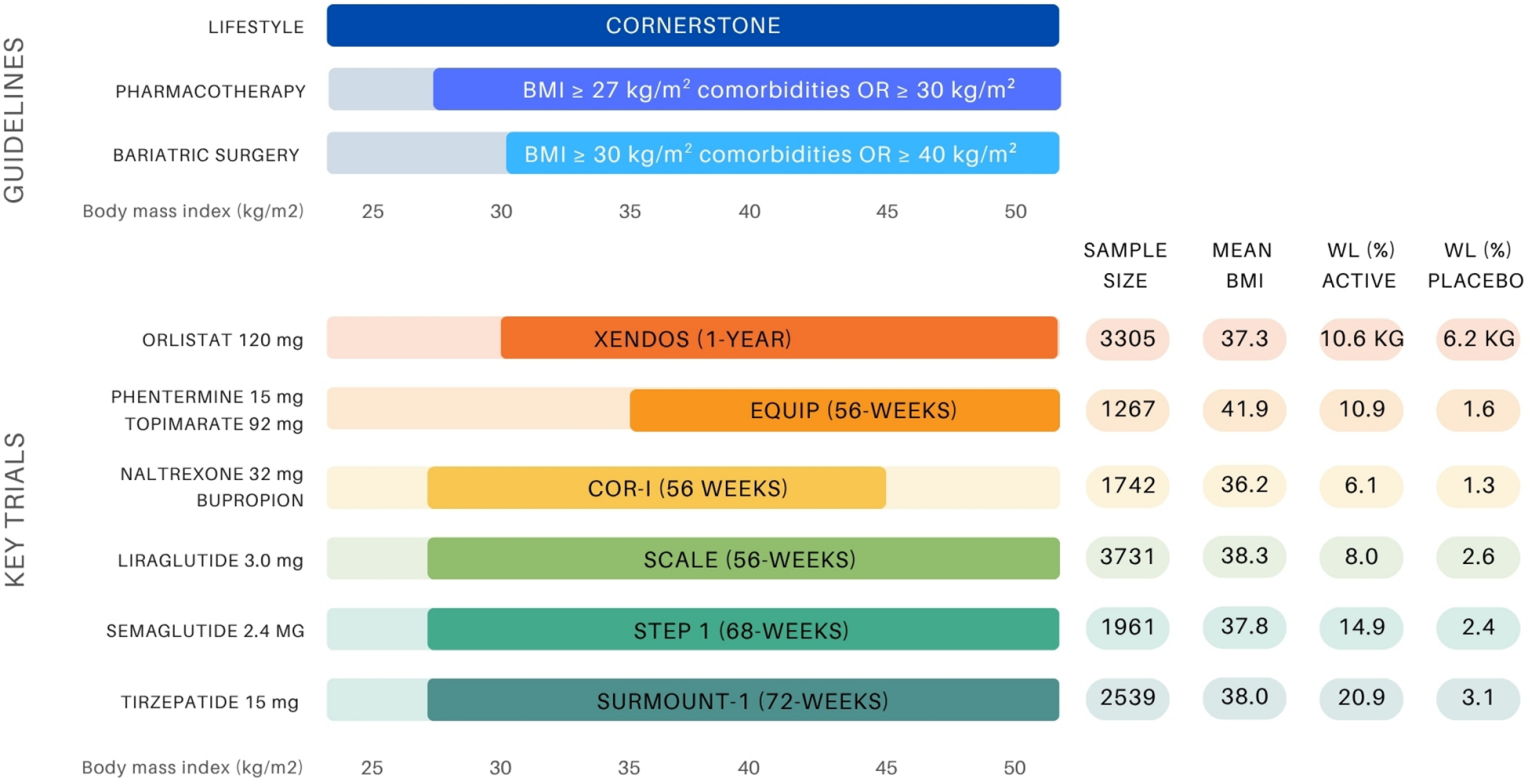
Overview of BMI-based treatment recommendations for obesity management and landmark anti-obesity drug trials (10, 11, 14, 19-21, 23, 25, 34, 35). The upper panel presents guideline thresholds for lifestyle intervention, pharmacotherapy (BMI ≥27 kg/m² with comorbidities or ≥30 kg/m²), and bariatric surgery (BMI ≥35–40 kg/m² depending on comorbidity status). The lower panel displays key clinical trials supporting the approval of six anti-obesity agents, indicating sample size, mean baseline BMI (entire group or active group), and weight loss outcomes (%WL unless otherwise stated) in both the active and placebo arms.

**Figure 2:**
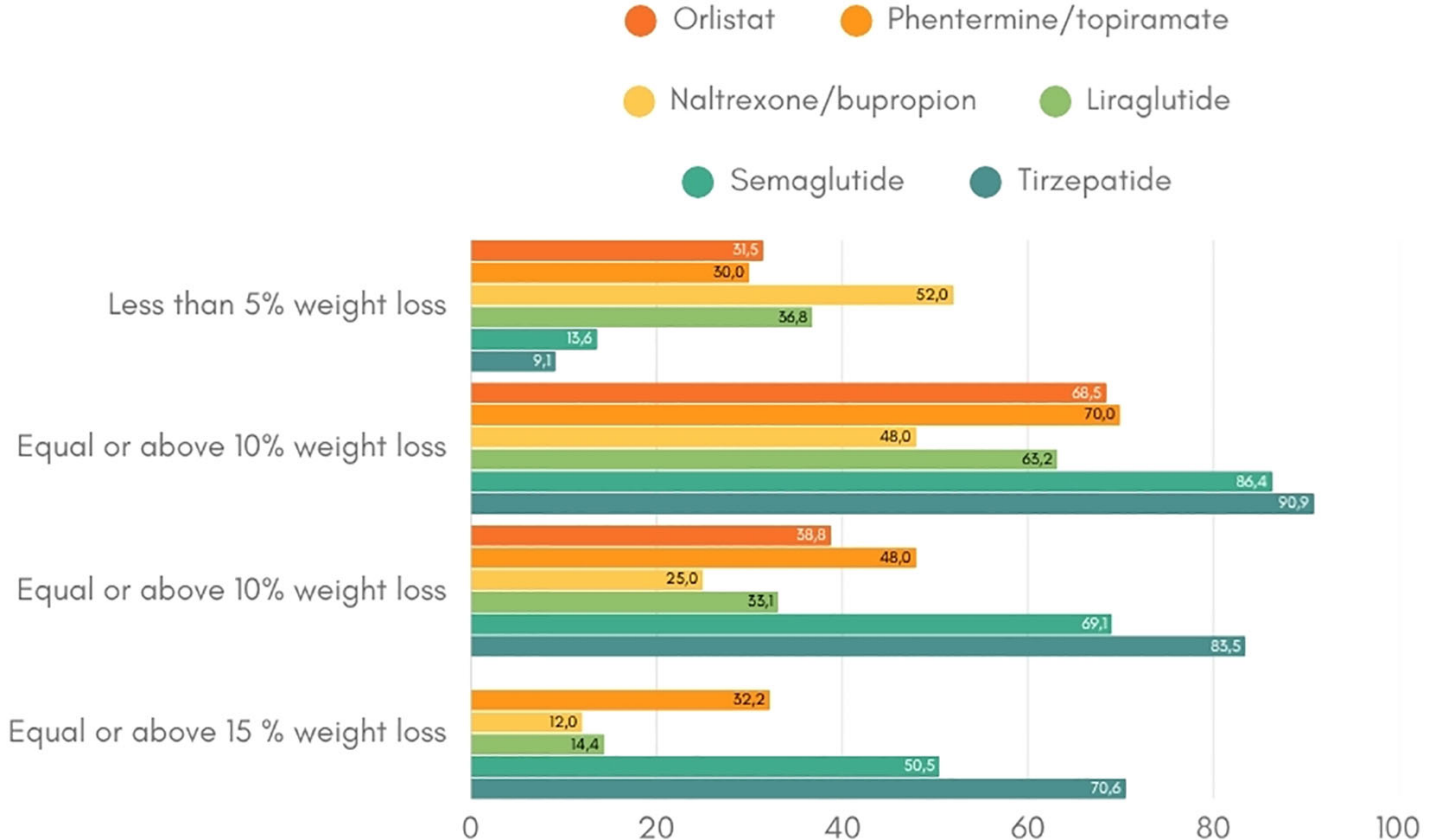
Weight loss response per anti-obesity drug (19-21, 23, 25, 34, 35). This figure indicates the percentage of patients that achieve the weight loss targets of at least 5%, 10% and 15% per anti-obesity medication.

**Figure 3:**
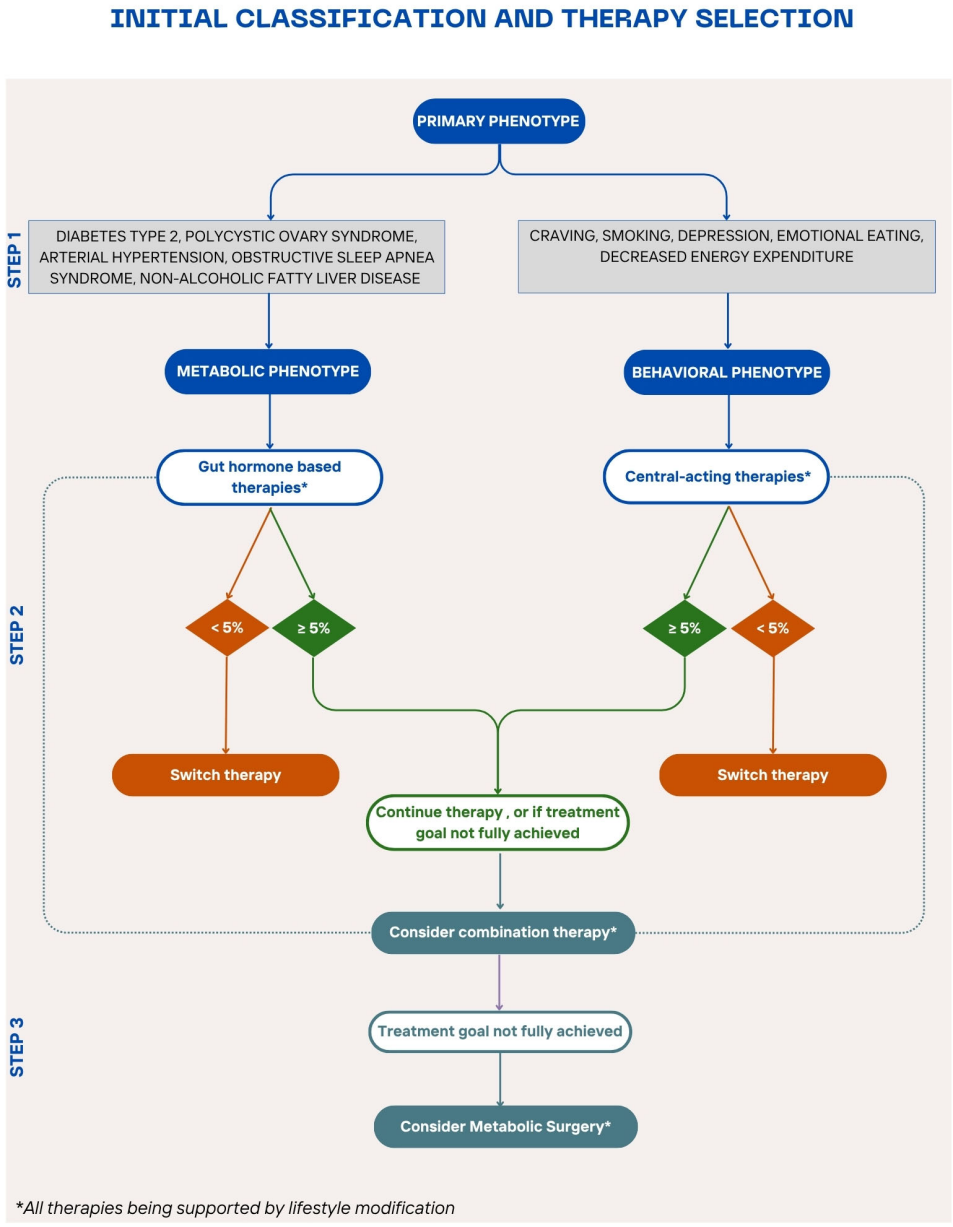
Stepwise algorithm for personalized obesity pharmacotherapy A proposed treatment algorithm integrating patient phenotypes, comorbidities, and treatment responses to guide personalized obesity management for patients who do not meet the criteria for metabolic surgery or have contraindications or not open to this option. The algorithm begins with lifestyle modification as the foundation, followed by phenotype-driven pharmacotherapy selection—gut hormone-based therapy for metabolic comorbidities and centrally acting therapy for behavioral/psychological factors. Treatment response is evaluated after 3 months to determine the need for therapy adjustment, combination treatment, or escalation to bariatric surgery for non-responders.

The original version of this article has been updated.

